# Association between a laboratory-based frailty index and mortality of critically ill patients with acute pancreatitis: a retrospective study

**DOI:** 10.3389/fnut.2025.1519112

**Published:** 2025-04-28

**Authors:** Zhichao Zou, Zhe Li, Qihai Wan, Qianqian Wang, Yi Yu

**Affiliations:** ^1^Department of Anesthesiology, The First Hospital of Hunan University of Chinese Medicine, Changsha, Hunan, China; ^2^Department of Intensive Care Medicine, Juancheng County People's Hospital, Heze, Shandong, China; ^3^Department of Anesthesiology, West China Second University Hospital, Sichuan University, Chengdu, Sichuan, China; ^4^Key Laboratory of Birth Defects and Related Diseases of Women and Children, Sichuan University, Ministry of Education, Chengdu, Sichuan, China; ^5^Department of Pulmonary and Critical Care Medicine, The First Affiliated Hospital, Sun Yat-sen University, Guangzhou, Guangdong, China; ^6^Department of Critical Care Medicine, The Second Affiliated Hospital of Guangzhou University of Chinese Medicine, Guangzhou, Guangdong, China

**Keywords:** acute pancreatitis, Medical Information Mart for Intensive Care-IV database, frailty index derived from laboratory tests, multivariate Cox regression, propensity score matching

## Abstract

**Background:**

Acute pancreatitis (AP) is associated with significant global mortality and morbidity. Frailty, which can be assessed through clinical indicators and life history, is known to impact adverse outcomes across different medical conditions. The frailty index derived from laboratory tests (FI-Lab) is a novel approach to the quantification of frailty. This study sought to investigate the relationship between the FI-Lab and mortality among critically ill patients with AP.

**Methods:**

We utilized data on patients diagnosed with AP from the Medical Information Mart for Intensive Care-IV database. The FI-Lab was calculated using a specific set of laboratory parameters indicative of physiological disturbances. The primary outcomes examined were 30-day and 90-day mortality rates. Multivariate Cox regression was used for the statistical analysis, with adjustments for age, gender, Acute Physiology and Chronic Health Evaluation II scores, and other variables. Propensity matching scores were used to ensure the robustness of our findings.

**Results:**

A total of 1,116 AP patients were included in the analysis (mean age = 58.4 years; 57.9% male). Each 0.1 increment of FI-Lab was found to increase the risks of 30-day and 90-day mortality by 30% (hazard ratio (HR) = 1.30, *p* < 0.001 for both). The propensity score matching (PSM) analysis validated these results. The FI-Lab demonstrated an association with acute kidney injury and the requirement for continuous renal replacement therapy. However, these associations were not significant after the PSM analysis.

**Conclusion:**

An elevated FI-Lab was associated with higher mortality rates among critically ill AP patients. Randomized controlled trials are needed to confirm these findings and to explore their clinical implications.

## Introduction

Acute pancreatitis (AP) is one of the most prevalent gastrointestinal emergencies in clinical practice, and its global incidence is rising. Annually, it accounts for over 288,220 hospital admissions and contributes to costs exceeding $2.2 billion in the United States alone (1). The clinical presentations of AP range from mild, self-limiting conditions to severe cases characterized by multi-organ failure, systemic inflammatory response syndrome, and the need for admission to an intensive care unit (ICU) to provide close monitoring and aggressive treatment (2, 3). Assessing the prognosis of critically ill patients with AP remains a significant challenge in both clinical and scientific contexts. Traditional tools for assessment, such as the Acute Physiology and Chronic Health Evaluation, Ranson’s Criteria, Modified Glasgow Score, Systematic Inflammatory Response Syndrome Score, and the Bedside Index for Severity of AP (BISAP), have demonstrated limitations in their ability to predict short- and long-term mortality, particularly in contexts pertaining to the prognosis of the individual (4, 5). Therefore, there is an urgent need for a new and more accurate tool to predict the prognosis of patients with severe acute pancreatitis, in order to provide a scientific basis for clinical decision-making.

The concept of frailty was introduced to critical care medicine following in-depth studies on the pathophysiological mechanisms of critically ill patients (6). Frailty is associated with advanced age, chronic disease, malnutrition, and multisystem functional decline, reflecting a reduced physiological reserve capacity to cope with stressors (7). It is a biological syndrome resulting from decreased physiological reserves of multiple organs (8). Over the past decade, substantial research has demonstrated the utility of frailty status as a prognostic indicator for adverse events and complications related to various clinical treatments, including pancreatoduodenectomies, pancreatic resections, kidney transplants, percutaneous coronary interventions, and colectomies (9–14). Notably, frailty status remains under-explored as a predictor in patients with AP.

Timely recognition of frailty is challenging, requiring specialized tools and face-to-face assessments such as grip strength measurements. The frailty index based on laboratory tests (FI-Lab) proposed by Howlett et al. consists of several objective laboratory tests and vital signs that can be constructed easily through routine clinical practice (15). The FI-Lab has demonstrated good diagnostic accuracy and the ability to predict clinical outcomes in diverse patient populations (16–21). In such patient populations, the FI-Lab may be more appropriate for assessing frailty in ICU patients with AP.

The objective of this study was to calculate the FI-Lab and investigate its relationship with patients’ prognoses by analyzing clinical data from ICU patients with AP from the Medical Information Mart for Intensive Care-IV (MIMIC-IV 3.0) database (2008–2022). We hypothesized that there would be a strong correlation between the FI-Lab and the clinical outcomes of patients with severe AP. Furthermore, we also hypothesized that patients with severe AP and higher FI-Lab scores would have longer ICU stays and a higher risk of death. Hence, this study aimed to provide new perspectives and tools to assess the prognoses of AP in critically ill patients, thereby informing clinical decision-making.

## Materials and methods

We selected patients with AP from the MIMIC-IV database (version 3.0), which contained 546,028 inpatient records and 94,458 ICU records of patients treated at the Beth Israel Deaconess Medical Center in Boston, United States, from 2008 to 2022 (22). One of the study’s co-authors, Yi Yu, had authorized access to the database (certificate ID: 6477678). The research process followed the Strengthening the Reporting of Observational Studies in Epidemiology (STROBE) guidelines (23).

### Study population and data extraction

The subjects of this study were patients diagnosed with AP, based on the International Classification of Diseases (ICD)-9 and ICD-10 codes. We focused only on patients with AP who were admitted to the ICU for the first time. Patients were excluded from the study based on the following criteria: (i) age <18 years, (ii) ICU stay <24 h, (iii) primary diagnosis not AP with more than 3 diagnostic codes, or (iv) missing more than 12 data points required to analyze the FI-Lab. We collected information on patients’ demographics, vital signs, laboratory results, comorbidities, clinical severity scores, and other relevant characteristics.

### Construction of the FI-lab

The FI-Lab was obtained using 33 items, including 30 laboratory test results (obtained from 24 h before to 48 h after the first ICU admission). These results included the findings of blood tests (white blood cell and platelet counts; hemoglobin, total bilirubin, alanine transaminase, albumin, alkaline phosphatase, lactate dehydrogenase, urea nitrogen, creatinine, glucose, potassium, sodium, calcium, phosphorus, fibrinogen, and troponin T levels; plasminogen time; international normalized ratio; and activated partial thromboplastin time), arterial blood gas analyses (pH, partial pressure of oxygen, partial pressure of carbon dioxide, and lactate levels), urinalysis (leukocyte and erythrocyte counts and the levels of protein, glucose, ketone bodies, and bilirubin), and the following three vital signs (averaged over the first day in the ICU): systolic blood pressure, diastolic blood pressure, and heart rate. Each item was dichotomized based on the normal reference ranges provided in the database: values within the reference interval were assigned a score of 0, and values outside the reference interval were assigned a score of 1. The reference values for each item are detailed in the Supplementary Table S1. FI-Lab values were calculated by summing the scores for all items and dividing the sum by the total number of items. Thus, the FI-Lab ranged from 0 to 1.

We used a generalized additive model to identify the nonlinear relationship. If a nonlinear correlation was observed, a two-piecewise linear regression model was conducted to calculate the threshold effect of the FI-lab on mortality in terms of the smoothing plot.

### Covariates

In addition to the indicators used to calculate the FI-Lab, we collected demographic and admission data, including age, sex, race, body mass index (BMI), marital status, insurance type, BISAP, Simplified Acute Physiology Score II (SAPS II), and Sequential Organ Failure Assessment (SOFA) scores. Comorbidity information included myocardial infarction, congestive heart failure, cerebrovascular disease, diabetes, renal disease, malignancy, severe liver disease, and sepsis. We also recorded patient interventions during their ICU stays, such as their use of mechanical ventilation, vasopressors, and continuous renal replacement therapy (CRRT). Multicollinearity was assessed using variance inflation factor analysis, with a variance inflation factor >2 as indicative.

### Outcomes

The primary outcomes of interest were the 30-day and 90-day mortality rates of patients with AP. Secondary outcomes included ICU and hospital-stay duration, acute kidney injury (AKI), and CRRT.

### Statistical analysis

The baseline characteristics of the patients were analyzed by group (low FI-Lab and high FI-Lab). Categorical data are presented as numbers (percentages), while continuous data were presented as means ± standard deviation or medians (interquartile ranges), as appropriate. Statistical analyses, including analysis of variance (ANOVA) or rank-sum tests were used to assess group differences in the continuous variables. We used the chi-square test or Fisher’s exact test to evaluate group differences in categorical variables.

The proportion of missing covariate data was less than 1% in all of the analyses. We imputed missing values for covariates using the median. The relationship between the FI-Lab and mortality risk was described using restricted cubic spline (RCS) curves. We conducted multivariate Cox regression analyses to evaluate the independent association between the FI-Lab and mortality, using adjusted models to account for the various covariates; four models were used in the regression analyses. Furthermore, we conducted logistic regression analyses to assess the risk of AKI and CRRT, along with linear regression analyses to evaluate ICU and hospital lengths of stay. Further analyses were adjusted for relevant covariates, subgroup analyses, and interaction analyses, comparing differences in the FI-Lab (as a continuous variable) between the survival and non-survival groups. To enhance the reliability of the results, we performed propensity score matching (PSM) to balance the baseline characteristics between the two groups, using a 1:1 nearest neighbor matching algorithm with a caliper of 0.2.

We evaluated the hospitalization survival rates based on the FI-Lab groups using Kaplan–Meier survival curves and assessed them using the log-rank test. Stratified and interaction analyses were performed based on age (<65 years or ≥ 65 years), sex (male or female), race (White or other), BMI (<25 or ≥ 25), diabetes (none, without complications, with complications), SOFA score of <4 or ≥4 and the BISAP severity score of <3 or ≥3.

All statistical analyses were conducted using STATA software (version 17.0), R software packages (http://www.R-project.org, R Foundation), and Free Statistics software version 1.8 (24). Statistical significance was considered at *p* < 0.05 (two-tailed).

## Results

### Participants

**Figure 1:**
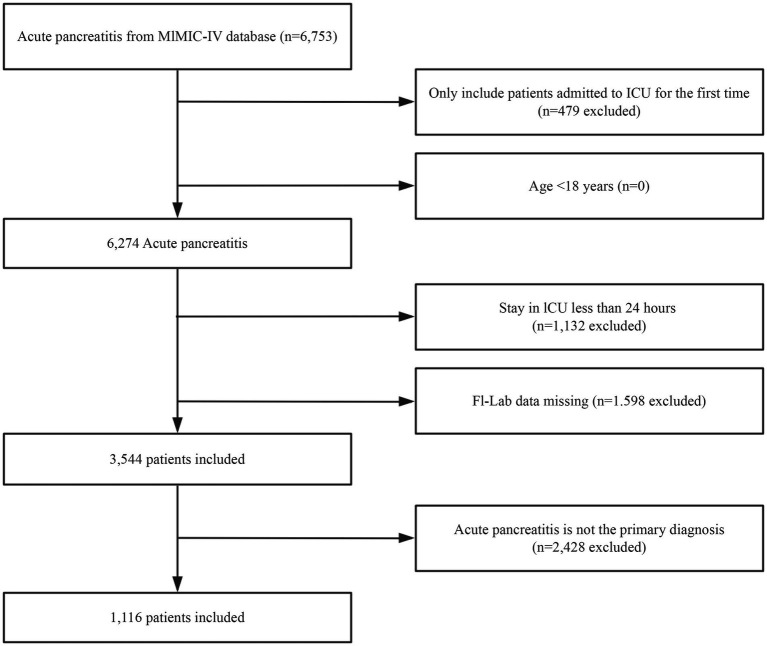
Flowchart outlining the process for enrollment of study participants.

A total of 6,753 patients met the diagnostic criteria for AP. After removing participants with repeated ICU admissions, patients under the age of 18, patients for which AP was not the primary diagnosis, and those with an ICU stay of less than 24 h, the final cohort consisted of 1,116 patients (The baseline information for the excluded populations due to missing data is illustrated in Supplementary Table S2). The selection process for the study participants is illustrated in Figure 1.

### Linear relationship between the FI-lab and mortality

**Figure 2:**
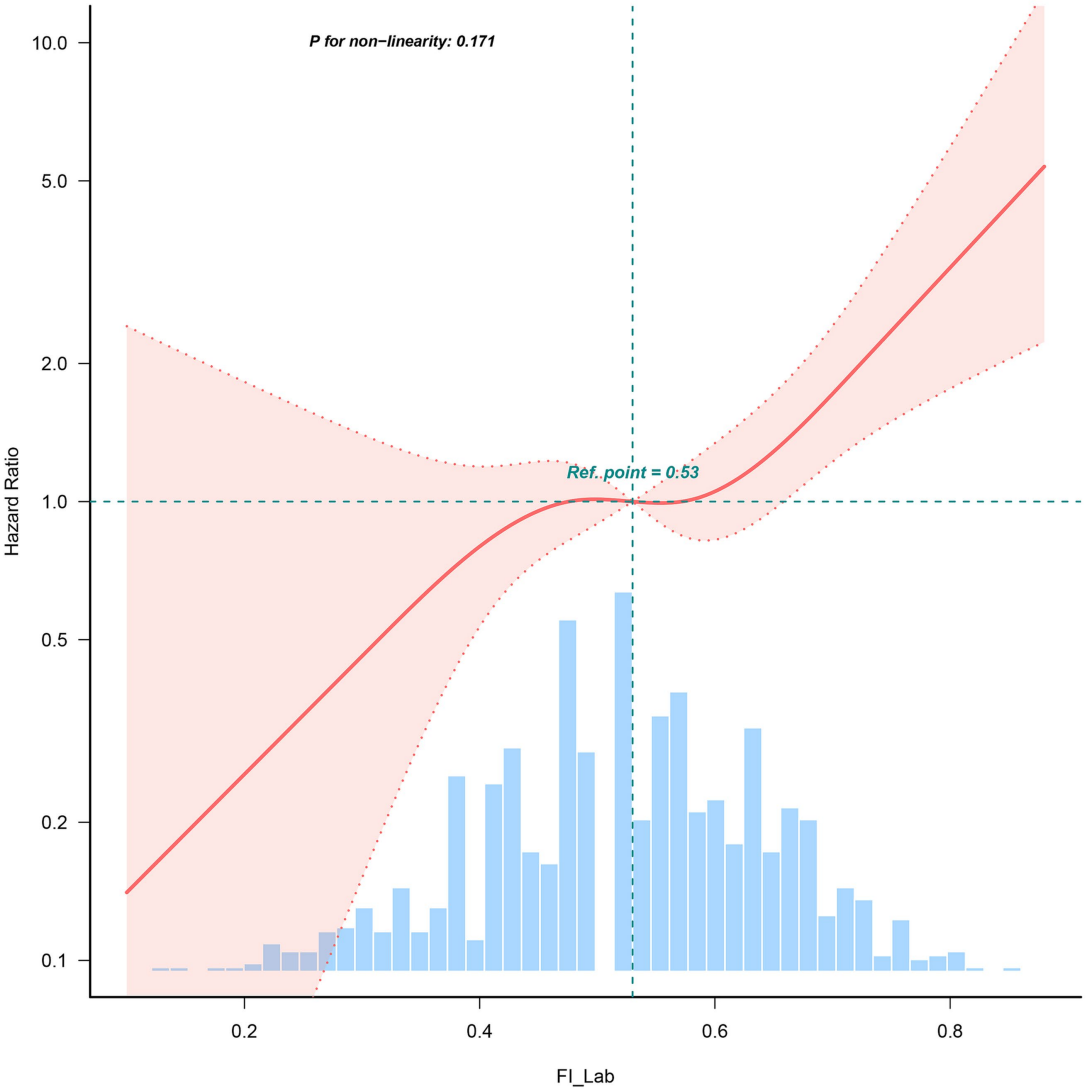
Spline curves showing the association of the FI-Lab as a continuous variable with 30-day mortality. The spline curves were adjusted for all factors of model 4 in multivariable Cox regression.

Restricted Cube Spline analysis revealed a linear relationship between the FI-Lab during ICU admission and the risk of mortality during the hospitalization of patients with AP (*P* for non-linearity: 0.171). Specifically, when the FI-Lab was 0.53, its Hazard Ratio (HR) was 1. Overall, with an increase in the FI-Lab, the risk of mortality in AP patients increased accordingly, as illustrated in Figure 2.

### Baseline characteristics

This study included 1,116 patients with a mean age of 58.4 ± 17.2 years, of whom 57.9% were men. Table 1 presents the baseline characteristics of the patient population. Comparatively, the low FI-Lab group tended to be younger, with lower SAPS-II and SOFA scores, vasoactive drug usage, sepsis, ventilation rates, and Rapid Response Team rates.

**Table 1:** Baseline characteristics of participants.

		Unmatched patients			Propensity-score–matched patients		
Variables	Total (*n* = 1,116)	Low Fi-lab (*n* = 573)	High Fi-lab (*n* = 543)	SMD	Low Fi-lab (*n* = 350)	High Fi-lab (*n* = 350)	SMD
Age, y	58.4 ± 17.2	57.09 ± 17.26	59.88 ± 17.07	>0.1	59.24 ± 16.97	59.67 ± 17.39	<0.1
Sex, male, *n* (%)	646 (57.9)	345 (60.2)	301 (55.4)	<0.1	204 (58.3)	201 (57.4)	<0.1
BMI, kg/m^2^	28.9 ± 5.4	28.89 ± 5.37	28.97 ± 5.48	<0.1	28.85 ± 5.04	28.97 ± 4.95	<0.1
Race, *n* (%)				<0.1			<0.1
White	667 (59.8)	333 (58.1)	334 (61.5)		216 (61.7)	206 (58.9)	
Others	449 (40.2)	240 (41.9)	209 (38.5)		134 (38.3)	144 (41.1)	
Marital status, *n* (%)				<0.1			<0.1
Married	480 (43.0)	244 (42.6)	236 (43.5)		154 (44.0)	148 (42.3)	
Unmarried	636 (57.0)	329 (57.4)	307 (56.5)		196 (56.0)	202 (57.7)	
Insurance, *n* (%)				<0.1			<0.1
Medicaid	236 (21.1)	129 (22.5)	107 (19.7)		78 (22.3)	75 (21.4)	
Medicare	507 (45.4)	257 (44.9)	250 (46)		160 (45.7)	170 (48.6)	
Private	317 (28.4)	157 (27.4)	160 (29.5)		96 (27.4)	87 (24.9)	
Others	56 (5.0)	30 (5.2)	26 (4.8)		16 (4.6)	18 (5.1)	
BISAP, *n* (%)							
≥3	568 (50.9)	235 (41)	333 (61.3)	>0.1	281 (49.4)	273 (50.3)	<0.1
Sepsis, *n* (%)	763 (68.4)	341 (59.5)	422 (77.7)	>0.1	251 (71.7)	247 (70.6)	<0.1
Saps-ii	36.2 ± 16.0	31.2 ± 12.9	41.4 ± 17.2	>0.1	34.74 ± 13.18	35.99 ± 15.02	<0.1
SOFA score	6.0 ± 4.1	4.6 ± 3.4	7.5 ± 4.3	>0.1	5.61 ± 3.58	5.88 ± 3.50	<0.1
Charlson comorbidity index	4.7 ± 2.9	4.4 ± 2.8	5.1 ± 2.8	<0.1	4.67 (2.93)	4.77 (2.94)	<0.1
Hypertension, *n* (%)	464 (41.6)	252 (44)	212 (39)	<0.1	137 (39.1)	146 (41.7)	<0.1
MI, *n* (%)	106 (9.5)	51 (8.9)	55 (10.1)	<0.1	55.1 (9.7)	53.5 (9.8)	<0.1
CHF, *n* (%)	199 (17.8)	99 (17.3)	100 (18.4)	<0.1	103.4 (18.1)	105.2 (19.3)	<0.1
CBVD, *n* (%)	48 (4.3)	21 (3.7)	27 (5)	<0.1	23.9 (4.2)	23.3 (4.3)	<0.1
CPD, *n* (%)	219 (19.6)	127 (22.2)	92 (16.9)	<0.1	107.4 (18.8)	105.0 (19.3)	<0.1
Diabetes, *n* (%)				<0.1			<0.1
None	784 (70.3)	406 (70.9)	378 (69.6)		387.5 (67.9)	380.8 (70.0)	
Without complications	240 (21.5)	117 (20.4)	123 (22.7)		133.8 (23.5)	115.5 (21.2)	
With complications	92 (8.2)	50 (8.7)	42 (7.7)		49.2 (8.6)	47.8 (8.8)	
Renal disease, *n* (%)	211 (18.9)	96 (16.8)	115 (21.2)	>0.1	74 (21.1)	67 (19.1)	<0.1
Malignant cancer, *n* (%)	106 (9.5)	39 (6.8)	67 (12.3)	>0.1	30 (8.6)	28 (8.0)	<0.1
Severe liver disease, *n* (%)	103 (9.2)	30 (5.2)	73 (13.4)	>0.1	28 (8.0)	22 (6.3)	<0.1
AKI, *n* (%)	679 (60.8)	280 (48.9)	399 (73.5)	>0.1	216 (61.7)	217 (62.0)	<0.1
RRT, *n* (%)	87 (7.8)	9 (1.6)	78 (14.4)	>0.1	9 (2.6)	5 (1.4)	<0.1
Vasoactive drug, *n* (%)	348 (31.2)	134 (23.4)	214 (39.4)	>0.1	101 (28.9)	105 (30.0)	<0.1
Ventilation, *n* (%)	428 (38.4)	187 (32.6)	241 (44.4)	>0.1	129 (36.9)	133 (38.0)	<0.1

For each variable, mean ± standard deviation, median (interquartile range), or number (percentage) is reported, as appropriate. SMD, standardized mean difference; BMI, body mass index; BISAP, Bedside Index for Severity in Acute Pancreatitis; SAPS II, simplified acute physiology score II; SOFA, sequential organ failure assessment; MI, myocardial infarct; CHF, congestive heart failure; CBVD, cerebrovascular disease; CPD, chronic pulmonary disease; ICU, intensive care unit; AKI, acute kidney injury; RRT, renal replacement therapy.

### Relationship between the FI-lab and mortality

Significant associations of the FI-Lab with 30-day mortality (hazard ratio (HR) = 1.86, 95% CI: 1.63–2.12, *p* < 0.001) and 90-day mortality (HR = 1.85, 95% CI: 1.64–2.10, *p* < 0.001) were observed in the univariate analysis of mortality risk (Table 2).

**Table 2:** Univariate and multivariate Cox regression models of FI-Lab with mortality in patients with AP.

Variable	Unadjusted Model	Model 1	Model 2	Model 3	Model 4	PSM
	HR 95% CI	HR 95% CI	HR 95% CI	HR 95% CI	HR 95% CI	HR 95% CI
30-day mortality						
FI-Lab (per 0.1 score)	1.86 (1.63–2.12)	1.86 (1.63–2.13)	1.32 (1.13–1.53)	1.27 (1.09–1.49)	1.28 (1.09–1.52)	1.39 (1.11–1.73)
FI-Lab (as a categorical variable)						
<0.53	1 (Ref)	1 (Ref)	1 (Ref)	1 (Ref)	1 (Ref)	1 (Ref)
≥0.53	3.52 (2.51–4.92)	3.39 (2.42–4.75)	1.72 (1.19–2.48)	1.58 (1.09–2.29)	1.51 (1.03–2.2)	1.69 (1.07–2.67)
90-day mortality						
FI-Lab (per 0.1 score)	1.85 (1.64–2.10)	1.86 (1.63–2.11)	1.31 (1.14–1.51)	1.26 (1.09–1.47)	1.27 (1.09–1.48)	1.39 (1.13–1.72)
FI-Lab (as a categorical variable)						
<0.53	1 (Ref)	1 (Ref)	1 (Ref)	1 (Ref)	1 (Ref)	1 (Ref)
≥0.53	3.34 (2.44–4.57)	3.23 (2.36–4.42)	1.62 (1.15–2.29)	1.49 (1.06–2.11)	1.43 (1.01–2.04)	1.67 (1.08–2.57)

AP, acute pancreatitis; HR, hazard ratio; Ref, reference; CI, confidence interval; FI-Lab, physiological and laboratory-based frailty index; PSM, propensity score matching.

Model 1: age, sex, BMI.

Model 2: Model 1, insurance, marital status, BISAP, saps ii, sofa.

Model 3: Model 2, AKI*, CRRT*, Ventilation*, vasoactive drug*. *:at any moment during ICU stay.

Model 4: Model 3, Charlson comorbidity index, hypertension, sepsis, myocardial infarction, congestive heart failure, cerebrovascular disease, chronic pulmonary disease, diabetes, renal disease, malignant cancer, severe liver disease.

PSM: Model 4.

Subsequently, in the extended multivariate Cox regression analysis (Table 2), the HRs of the FI-Labs remained consistently significant across all models for both the 30-day (HR range 1.30–1.86, *p* < 0.001 for all) and 90-day (HR range 1.28–1.86, *p* < 0.001 for all) mortality. After adjusting for all the covariates in Table 2, a 30% increase in the 30-day mortality risk (HR = 1.30, 95% CI: 1.10–1.53, *p* < 0.001, model 4) and a 30% increase in the 90-day mortality risk (HR = 1.45, 95% CI: 1.11–1.52, *p* < 0.001, model 4) were observed. The results from these models were robust.

We likewise did Area Under the Receiver Operating Characteristic curve (AUROC) analyses to clarify the ability of FI-Lab to distinguish survivors from non-survivors. This information will provide valuable insights into the value of FI-Lab in clinical practice (Supplementary Figure S1).

### Kaplan–Meier survival curve analysis

**Figure 3:**
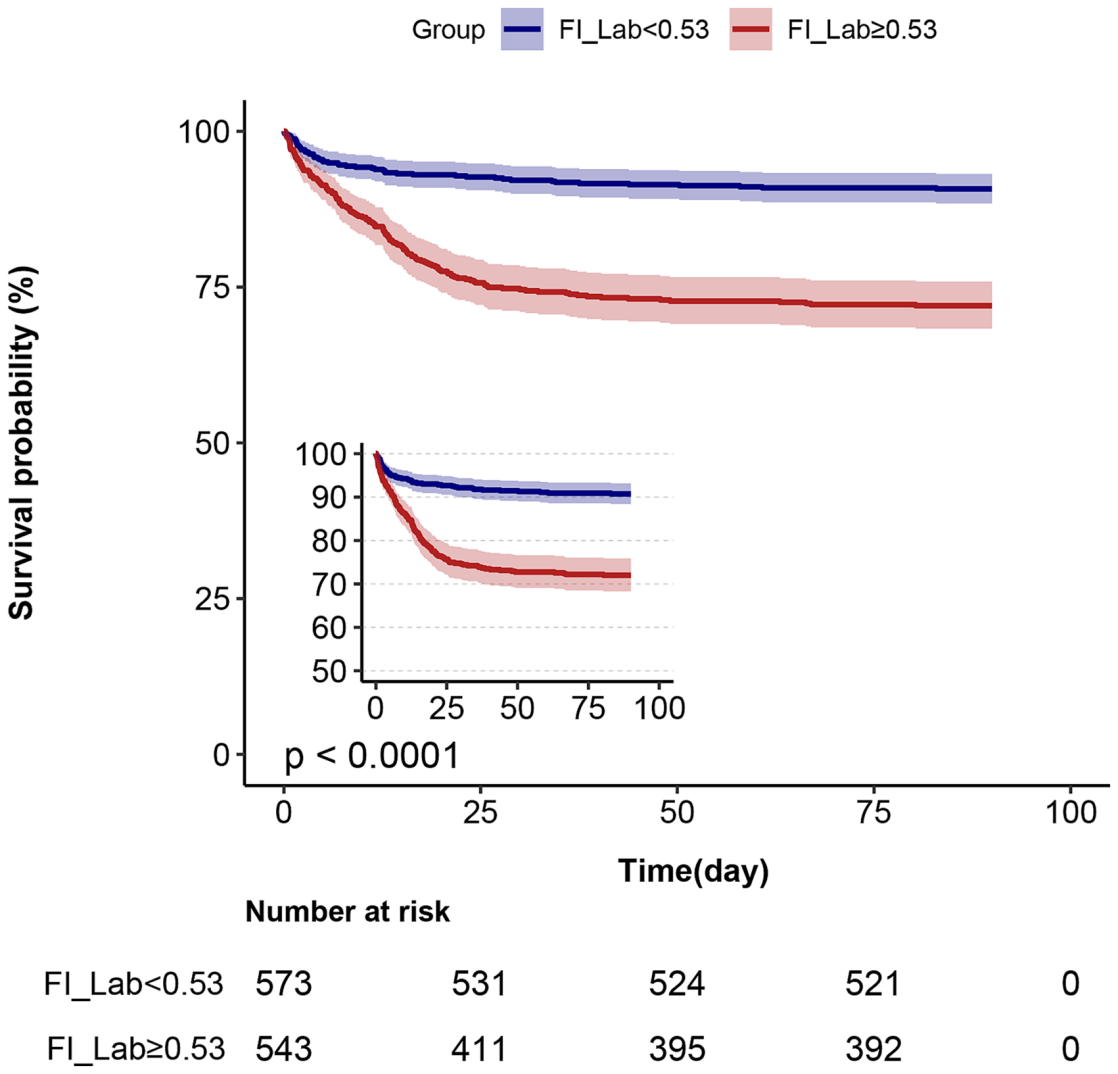
Kaplan–Meier survival curves, categorized by the FI-Lab, for patients with acute pancreatitis at day 90.

The Kaplan–Meier survival curves are presented in Figure 3. After grouping patients into two FI-Lab categories, the cumulative survival time during hospitalization was significantly lower as the FI-Lab increased (log-rank test, *p* < 0.001).

### Associations of the FI-lab with ICU and hospital stays

After incorporating all of the covariates listed in Table 3 into our models, no association was observed between patients’ ICU length of stay and FI-Lab levels when the FI-Lab increased (per 0.1) (*β* (95% CI) = −0.01 (−0.31 to 0.29)). Similarly, no association was found between length of stay and increasing FI-Lab (per 0.1) [*β* (95% CI) = 0.32 (−0.51 to 1.15)]. The same results were obtained when the FI-Lab was treated as a dichotomous variable, i.e., no significant differences found between the high and low FI-Lab groups [*β* (95% CI) = 0.21 (−0.51 to 0.92) and 0.42 (−1.55 to 2.39), respectively; Table 3].

**Table 3:** Associations of the FI-Lab with ICU stay and hospital stay among AP.

Variable	Length of stay in the ICU (days)			Length of stay in the hospital (days)		
	Model 1	Model 2	PSM	Model 1	Model 2	PSM
	*β* (95%CI)	*β* (95%CI)	*β* (95%CI)	*β* (95%CI)	*β* (95%CI)	*β* (95%CI)
FI-Lab (per 0.1 score)	0.95 (0.64–1.26)	−0.01 (−0.31 to 0.29)	0.18 (−0.16 to 0.52)	2.02 (1.27–2.76)	0.32 (−0.51 to 1.15)	0.35 (−0.65 to 1.36)
FI-Lab (as a categorical variable)						
<0.53	0 (Ref)	0 (Ref)	0 (Ref)	0 (Ref)	0 (Ref)	0 (Ref)
≥0.53	1.95 (1.18–2.72)	0.21 (−0.51 to 0.92)	0.24 (−0.5 to 0.97)	3.72 (1.85–5.59)	0.42 (−1.55 to 2.39)	0.26 (−1.91 to 2.44)

AP, acute pancreatitis; ICU, intensive care unit; Ref, reference; CI, confidence interval; FI-Lab, physiological and laboratory-based frailty index; PSM, propensity score matching.

Model 1: unadjusted.

Model 2: age, sex, BMI, insurance, marital status, BISAP, SAPS II, SOFA, AKI, RRT, Ventilation, vasoactive drug, Charlson comorbidity index, hypertension, sepsis, myocardial infarction, congestive heart failure, cerebrovascular disease, chronic pulmonary disease, diabetes, renal disease, malignant cancer, severe liver disease.

PSM: Model 2.

As seen in Table 4, the risk for developing AKI on day 7 and CRRT requirements were 33 and 68% higher, respectively, with a 0.1 increase in FI-Lab [odds ratios (ORs; 95% CIs) = 1.33 (1.16–1.52) and 1.68 (1.22–2.3), respectively]. The same results were obtained when the FI-Lab was treated as a dichotomous variable, with a higher risk for AKI observed in the high FI-Lab group than in the low FI-Lab group [ORs; 95% CIs = 1.83 (1.34–2.48) and 4.69 (1.98–11.12), respectively].

**Table 4:** Associations of the use of FI-Lab and AKIs and CRRTs among AP.

Variable		AKI			CRRT	
	Model 1	Model 2	PSM	Model 1	Model 2	PSM
	OR (95%CI)	OR (95%CI)	OR (95%CI)	OR (95%CI)	OR (95%CI)	OR (95%CI)
FI-Lab (per 0.1 score)	1.73 (1.55–1.93)	1.33 (1.16–1.52)	1.05 (0.9–1.23)	2.79 (2.21–3.53)	1.68 (1.22–2.3)	0.98 (0.46–2.09)
FI-Lab (as a categorical variable)						
<0.53	1 (Ref)	1 (Ref)	1 (Ref)	1 (Ref)	1 (Ref)	1 (Ref)
≥0.53	2.9 (2.26–3.73)	1.83 (1.34–2.48)	0.98 (0.69–1.39)	10.51 (5.22–21.18)	4.69 (1.98–11.12)	0.71 (0.15–3.36)

AP, acute pancreatitis; ICU, intensive care unit; Ref, reference; CI, confidence interval; FI-Lab, physiological and laboratory-based frailty index; PSM, propensity score matching.

Model 1: unadjusted.

Model 2: age, sex, BMI, insurance, marital status, BISAP, SAPS II, SOFA, ventilation, vasoactive drug, Charlson comorbidity index, hypertension, sepsis, myocardial infarction, congestive heart failure, cerebrovascular disease, chronic pulmonary disease, diabetes, renal disease, malignant cancer, severe liver disease.

PSM: Model 2.

### Subgroup and sensitivity analyses

**Figure 4:**
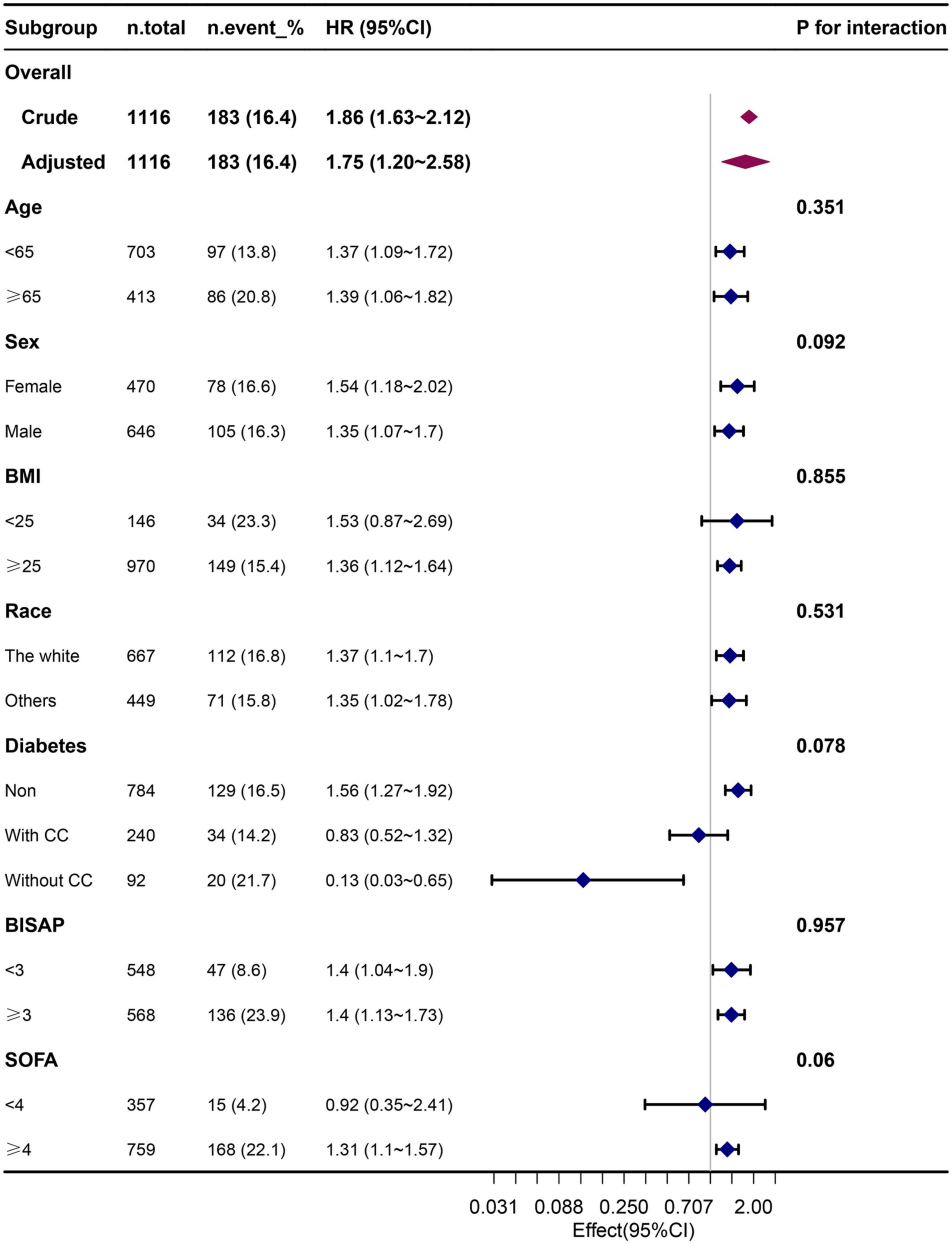
Association between the FI-Lab and 90-day mortality in relation to the baseline characteristics.

The results of our subgroup analysis indicated that the relationship between the FI-Lab and mortality remained robust and reliable, with no significant interaction observed in the subgroups (*p*-value for the interaction >0.05) (Figure 4).

After conducting the PSM, the two groups consisted of 350 well-matched pairs (*n* = 700), with no significant differences found in the key indicators between the matched groups (Table 1).

Our findings remained robust across the Cox regression models in Table 2, showing a 48% increase in the 30-day mortality risk (per 0.1) (HR = 1.48, 95% CI: 1.17–1.87, *p* < 0.001) and a 37% increase in the 90-day mortality risk (per 0.1) (HR = 1.37, 95% CI: 1.16–1.62, *p* < 0.001). When treating the FI-Lab as a categorical variable, the HR of the FI-Lab remained consistently significant across all models (HR range 1.72–3.52, *p* < 0.001 for all) for 30-day delirium. A similar trend was observed for 90-day delirium (Table 2). This analysis also showed no significant association between the ICU and hospital length of stay with FI-Lab (Table 3). Moreover, this analysis found that the risk for AKI on day 7 and CRRT requirements were not significantly associated with the FI-Lab, whether it was treated as a continuous or categorical variable (Table 4).

## Discussion

### Main findings

Our study presents one of the most comprehensive cohort-analyses of the association of the FI-Lab with the mortality of ICU patients with AP. We observed that an elevated FI-Lab level significantly increased the risk of 30-day and 90-day mortality, and these findings remained robust across various analyses, including those that adjusted for confounders using PSM. Subgroup analyses and Kaplan–Meier curves further corroborated these trends. The RCS curve analysis found a linear relationship between the FI-Lab and mortality risk, indicating that higher FI-Lab levels were associated with increased mortality. This finding suggests that the FI-Lab serves as valuable prognostic markers, which can clinicians in assessing outcomes of ICU patients with AP. Overall, an elevated FI-Lab among ICU patients with AP may indicate a poor prognosis, supporting clinical decision-making and risk stratification.

### Effects of the FI-lab and mortality of critically ill AP patients

The novel frailty index, FI-Lab, has consistently demonstrated robust prognostic and predictive value across various disease states (16, 25, 26). Recent studies indicate that the FI-Lab can effectively predict in-hospital mortality among critically ill ICU patients, and its integration with other frailty measures may enhance the identification of patients with an elevated risk for in-hospital mortality (25). However, research on the application of FI-Lab specifically in ICU patients with AP remains limited. Our study addresses this gap, representing the first systematic investigation into the use of the FI-Lab with ICU patients who have AP.

The findings of our study are consistent with previous research, indicating that frailty is independently associated with increased mortality and readmission rates of patients with acute biliary pancreatitis, thereby reinforcing the prognostic significance of frailty indices in AP patients (27). One study highlights the utility of dynamic frailty assessments as a management tool in cases of necrotizing pancreatitis, suggesting that the FI-Lab may function similarly in AP scenarios (28). Our study corroborates findings that identify frailty as a factor associated with heightened mortality risk, complications, and increased healthcare costs among patients with AP (29). The frailty risk score serves as a valuable risk stratification tool for assessing the prognosis of AP patients, further emphasizing the relevance of the FI-Lab in the prognostic evaluation of this condition (30, 31).

To ensure robustness in our analysis, we employed PSM to adjust for confounding variables (32). An elevated FI-Lab was identified as a significant risk factor for increased mortality in patients with AP. More importantly, mortality increased with a higher FI-Lab, even after the adjustments for PSM and potential confounders.

It is critical to recognize that frailty is a reversible condition; it is potentially preventable and treatable. Therefore, identifying frailty in ICU patients is essential, along with implementing appropriate management strategies, such as encouraging physical activity and providing nutritional supplements. Such interventions could significantly enhance patient outcomes (33, 34).

### Associations of ICU and hospital lengths of stay with FI-lab levels

No significant association was observed between FI-Lab levels and the length of ICU or hospital stays. Regardless of whether the FI-Lab was analyzed as a continuous or dichotomous variable (high vs. low FI-Lab), none of the increases in the FI-Lab was associated with prolonged ICU or hospital stays.

It is essential to consider other potential confounders. The frail state of a patient may indirectly influence their recovery and management of complications, thereby affecting their hospital length of stay. For instance, frail patients might experience a delayed recovery due to impaired immune functioning or poor nutritional status, leading to an extended hospitalization (35–37). However, this indirect effect was not observed in our simple regression analysis, indicating a need for more complex multivariate models in future research to explore these relationships further.

### Associations between the incidence of AKI, CRRT requirements, and levels of FI-lab

In contrast to the findings related to the hospital and ICU stays, our results demonstrated a significant positive association of FI-Lab level, with AKI incidence and CRRT requirements. Specifically, an increase of 0.1 on the FI-Lab was associated with a 33% higher risk of AKI and a 68% higher risk of requiring CRRT. This finding aligns with previous research, suggesting that frailty is an important prognostic factor in critically ill patients, particularly those with renal dysfunction. For instance, studies have reported that frailty significantly elevates the risk of AKI among critically ill patients (38, 39). Another study found that frail patients are at a higher risk of experiencing AKI and require more CRRT following cardiac surgery (40). These findings corroborate our observations and underscore the predictive value of the FI-Lab for the necessary monitoring and treatment of AKI and CRRT. Thus, our study adds to the growing body of literature supporting frailty as an independent risk factor for AKI across various clinical contexts.

However, following the PSM analysis, we found no significant associations between the FI-Lab with AKI and CRRT requirements. This finding may suggest that selection bias or other confounding variables played a role in the results of the initial analyses. Nevertheless, this does not entirely rule out the potential impact of frailty on acute complications. Therefore, further studies should use larger sample sizes and additional covariates to validate these relationships.

### Strengths of our study

Our study has several notable strengths. First, we utilized a comprehensive and publicly accessible database, which enhances the reliability and comprehensiveness of our data. Second, to the best of our knowledge, no previous study has examined the specific impact of the FI-Lab on mortality risk in ICU patients diagnosed with AP. Our findings provide robust and conclusive evidence that the elevated FI-Lab levels are strongly associated with the poor prognoses observed in this patient population. Third, we conducted multiple regression and PSM analyses to ensure the robustness and reliability of our results. This rigorous analytical approach further strengthens the credibility and internal validity of our findings. Fourth, the FI-Lab is based on readily available laboratory indicators and vital signs; hence, it is relatively simple to calculate and has the potential for widespread clinical application. Clinicians can use the FI-Lab to identify high-risk groups among patients with severe AP at an early stage, thereby facilitating timely intervention and optimization of treatment strategies. For instance, patients with high FI-Lab levels may benefit from more aggressive monitoring and treatments, including frequent laboratory tests and enhanced organ function support.

### Limitations of our study

Although this study represents the most comprehensive investigation to date on the use of a FI-Lab for assessing and treating ICU patients with AP, it has several limitations. First, the FI-Labs of patients was measured only at the time of ICU admission; thus, their FI-Labs may have changed with the patient’s condition during hospitalization. Dynamic changes in the FI-Lab could be captured through continuous monitoring, and the prognostic significance of such changes in ICU patients with sepsis, remains unclear, warranting further exploration. Second, the generalizability of our findings may be limited as the study was conducted using data from a single ICU in the USA. Third, some factors known to influence AP mortality were not reported in the available studies, such as the timing of appropriate antibiotic administration, volume resuscitation, history of alcohol consumption, and phosphorus levels. Therefore, we could not analyze these potentially important confounders. Fourth, as this was not an experimental study, our research design prevented us from evaluating the causal effects of the FI-Lab. Fifth, FI-Lab is an objective, automated measure based on readily available laboratory values, making it feasible for large-scale implementation in ICU settings. That said, FI-Lab may not capture (functional, cognitive and other) aspects of frailty. Moreover, our study focused primarily on 30-day and 90-day mortality, without any long-term follow-ups to assess the utility of the FI-Lab in evaluating long-term prognoses. Randomized controlled trials would be better suited to address these questions. Nevertheless, some of these limitations are partially mitigated by the large sample size and use of the PSM to reduce confounding biases.

## Conclusion

This study demonstrates a strong association between elevated FI-Lab levels and poor prognoses of ICU patients with AP. The implementation of FI-Lab as a prognostic tool has the potential to aid in early risk stratification of high-risk patients upon ICU admission, thereby improving clinical outcomes. However, further studies with experimental designs (i.e., randomized controlled trials) are essential to validate these findings and confirm proposed hypotheses.

## Data Availability

The raw data supporting the conclusions of this article will be made available by the authors, without undue reservation.

## Supplementary material

SUPPLEMENTARY FIGURE S1 Comparison of predictive performance for 30-day mortality in ICU acute pancreatitis patients using FI-Lab, APACHE II, SOFA and BISAP scores. The curves illustrate the Receiver Operating Characteristic (ROC) analysis for each score, comparing sensitivity (true positive rate) against 1-specificity (false positive rate) for predicting 30-day mortality in ICU patients with acute pancreatitis. The area under the curve (AUC) values with their 95% confidence intervals for each score are annotated on the graph: FI-Lab: AUC = 0.707 (95% CI: 0.667 to 0.748); APACHE II: AUC = 0.771 (95% CI: 0.734 to 0.809); SOFA: AUC = 0.759 (95% CI: 0.722 to 0.796); BISAP: AUC = 0.678 (95% CI: 0.639 to 0.718).
