# Supplementary figures and images for: Association between a laboratory-based frailty index and mortality of critically ill patients with acute pancreatitis: a retrospective study
Source: Front Nutr. 2025 Apr 28;12:1519112. doi: 10.3389/fnut.2025.1519112 (PMC12066625; doi:10.3389/fnut.2025.1519112)

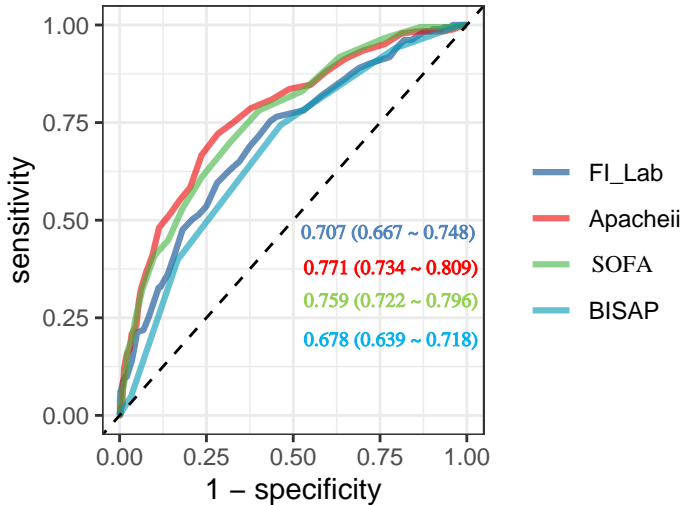

Supplement: SUPPLEMENTARY FIGURE S1 — Comparison of predictive performance for 30-day mortality in ICU acute pancreatitis patients using FI-Lab, APACHE II, SOFA and BISAP scores. The curves illustrate the Receiver Operating Characteristic (ROC) analysis for each score, comparing sensitivity (true positive rate) against 1-specificity (false positive rate) for predicting 30-day mortality in ICU patients with acute pancreatitis. The area under the curve (AUC) values with their 95% confidence intervals for each score are annotated on the graph: FI-Lab: AUC = 0.707 (95% CI: 0.667 to 0.748); APACHE II: AUC = 0.771 (95% CI: 0.734 to 0.809); SOFA: AUC = 0.759 (95% CI: 0.722 to 0.796); BISAP: AUC = 0.678 (95% CI: 0.639 to 0.718). [file Data_Sheet_1.PDF]
