# Supplementary material for: Association between a laboratory-based frailty index and mortality of critically ill patients with acute pancreatitis: a retrospective study
Source: Front Nutr. 2025 Apr 28;12:1519112. doi: 10.3389/fnut.2025.1519112 (PMC12066625; doi:10.3389/fnut.2025.1519112)
Supplement: Supplementary file 2 [file Table_1.docx]

**Table S1** Reference range of items used for construction of FI-Lab items

| Items | Reference range |
| --- | --- |
| Vital signs |  |
| Systolic blood pressure (mmHg) | 90-140 |
| Diastolic blood pressure (mmHg) | 60-90 |
| Heart rate (bpm) Blood samples | 60-99 |
| White cell count (×103/μL) | 4-11 |
| Platelet count (×109/L) | 150-440 |
| Hemoglobin (g/dL) | Female: 12-16  Male: 14-18 |
| Total bilirubin (mg/dL) | 0-1.5 |
| Alanine transaminase (Units/L) | 0-40 |
| Albumin (g/dL) | 3.5-5 |
| Alkaline phosphatase (Units/L) | 35-105 |
| Lactate dehydrogenase (Units/L) | 94-250 |
| Urea nitrogen (mg/dL) | 6-20 |
| Creatinine (mg/dL) | Female: 0.4-1.1  Male: 3.5-5.2 |
| Glucose (mg/dL) | 70-110 |
| Potassium (mmol/L) | 3.5-5.4 |
| Sodium (mmol/L) | 133-145 |
| Calcium (mg/dL) | 8.4-10.3 |
| Phosphorus (mg/dL) | 2.7-4.5 |
| Prothrombin time (s) | 9.4-12.5 |
| International normalized ratio | 0.9-1.1 |
| APTT (s) | 25-35 |
| Fibrinogen (mg/dL) | 150-400 |
| Troponin T (ng/mL) | 0-0.01 |
| Arterial blood gas samples |  |
| PH | 7.35-7.45 |
| PO2 (mmHg) | 85-105 |
| PCO2 (mmHg) | 35-45 |
| Lactate (mmol/L) Urine sample | 0.5-2 |
| Leucocytes | Negative |
| Erythrocytes | Negative |
| Protein | Negative |
| Glucose | Negative |
| Ketones | Negative |
| Bilirubin | Negative |

APTT, activated partial thromboplastin time; PH, potential of hydrogen; PO2, partial pressure of oxygen; PCO2, partial pressure of carbon dioxide
