# Supplementary material for: Association between a laboratory-based frailty index and mortality of critically ill patients with acute pancreatitis: a retrospective study
Source: Front Nutr. 2025 Apr 28;12:1519112. doi: 10.3389/fnut.2025.1519112 (PMC12066625; doi:10.3389/fnut.2025.1519112)
Supplement: Supplementary file 3 [file Table_2.docx]

**Table S2**: Baseline information table for excluded populations.

| **Variables** | **Total (n = 2,714)** | **Excluded (n = 1,598)** | **Included (n = 1,116)** | **P** |
| --- | --- | --- | --- | --- |
| Age, y | 61.4 ± 17.1 | 59.5 ± 16.6 | 58.4 ± 17.2 | >0.1 |
| Sex, Male, n (%) | 1,480 (54.5) | 834 (52.2) | 646 (57.9) | <0.05 |
| BMI, kg/m^2^ | 28.7 ± 6.0 | 28.4 ± 7.0 | 28.9 ± 5.4 | >0.05 |
| **Race, n (%)** |  |  |  | <0.05 |
| White | 1,692 (62.3) | 1,025 (64.1) | 667 (59.8) |  |
| Others | 1,022 (37.7) | 573 (35.9) | 449 (40.2) |  |
| **Marital status,** |  |  |  | <0.05 |
| Married, n (%) | 1,232 (45.4) | 752 (47.1) | 480 (43) |  |
| Unmarried, n (%) | 1,482 (54.6) | 846 (52.9) | 636 (57) |  |
| **Insurance, n (%)** |  |  |  | < 0.001 |
| Medicaid | 449 (16.5) | 213 (13.3) | 236 (21.1) |  |
| Medicare | 1,344 (49.5) | 837 (52.4) | 507 (45.4) |  |
| Private | 799 (29.4) | 482 (30.2) | 317 (28.4) |  |
| Others | 122 ( 4.5) | 66 (4.1) | 56 (5) |  |
| **BISAP, n (%)** |  |  |  |  |
| **≥3** | 1,404(51.7) | 836 (52.3) | 568 (50.9) | >0.1 |
| **Sepsis, n (%)** | 1,833 (67.5) | 1,070 (66.9) | 763 (68.4) | >0.1 |
| **Saps-ii** | 35.6 ± 14.0 | 35.1 ± 12.1 | 36.2 ± 16.0 | >0.1 |
| **SOFA score** | 5.4 ± 3.6 | 5.2 ± 2.6 | 6.0 ± 4.1 | >0.05 |
| **Charlson comorbidity index** | 4.8 ± 2.8 | 5.0 ± 2.8 | 4.7 ± 2.9 | >0.1 |
| **Hypertension, n (%)** | 1181 (43.5) | 717 (44.9) | 464 (41.6) | >0.1 |
| **MI, n (%)** | 302 (11.1) | 196 (12.3) | 106 (9.5) | <0.05 |
| **CHF, n (%)** | 514 (18.9) | 315 (19.7) | 199 (17.8) | >0.1 |
| **CBVD, n (%)** | 340 (12.5) | 292 (18.3) | 48 (4.3) | < 0.001 |
| **CPD, n (%)** | 554 (20.4) | 335 (20.9) | 219 (19.6) | >0.05 |
| **Diabetes, n (%)** |  |  |  | >0.05 |
| None | 1,996 (73.5) | 1,212 (75.8) | 784 (70.3) |  |
| Without complications | 514 (18.9) | 274 (17.1) | 240 (21.5) |  |
| With complications | 204 ( 7.5) | 112 (7) | 92 (8.2) |  |
| **Renal disease, n** **(%)** | 524 (19.3) | 313 (19.6) | 211 (18.9) | >0.1 |
| **Malignant cancer, n (%)** | 286 (10.5) | 180 (11.2) | 106 (9.5) | >0.05 |
| **Severe liver disease, n (%)** | 279 ( 10.3) | 1,76(11.0) | 103 (9.2) | >0.05 |
| **AKI, n (%)** | 1,607 (59.2) | 928 (58.1) | 679 (60.8) | >0.1 |
| **RRT, n (%)** | 224 ( 8.3) | 137 (8.6) | 87 (7.8) | >0.05 |
| **Vasoactive drug, n (%)** | 817 (30.1) | 469 (29.3) | 348 (31.2) | >0.1 |
| **Ventilation, n (%)** | 1048 (38.6) | 620 (38.8) | 428 (38.4) | >0.1 |

For each variable, mean ± standard deviation, median (interquartile range), or number (percentage) is reported, as appropriate.

Abbreviations: SMD, standardized mean difference; BMI, body mass index; BISAP, Bedside Index for Severity in Acute Pancreatitis; SAPS II, simplified acute physiology score II; SOFA, sequential organ failure assessment; MI, myocardial infarct; CHF, congestive heart failure; CBVD, cerebrovascular disease; CPD, chronic pulmonary disease; ICU, intensive care unit; AKI, acute kidney injury; RRT, renal replacement therapy.
